# Validation of Chinese medicine syndrome differentiation in early breast cancer: a multicenter prospective clinical study

**DOI:** 10.3389/fonc.2026.1652339

**Published:** 2026-02-02

**Authors:** Wei Luo, Yan Dai, Jiahua Wu, Xiaohong Xue, Lifang Liu, Weihe Bian, Xiaohong Xie, Gang Lyu, Ri Hong, Chang Qiu, Xiaojie Lin, Rui Xu, Qianqian Guo, Qianjun Chen

**Affiliations:** 1 State Key Laboratory of Traditional Chinese Medicine Syndrome/Breast Disease Specialist Hospital, The Second Affiliated Hospital of Guangzhou University of Chinese Medicine, Guangdong Provincial Hospital of Chinese Medicine, Guangdong Provincial Academy of Chinese Medical Sciences, Guangzhou, Guangdong, China; 2 Chinese Medicine Guangdong Laboratory, Guangzhou, Guangdong, China; 3 The Second Clinical College of Guangzhou University of Chinese Medicine, Guangzhou, Guangdong, China; 4 Breast Disease Specialist Hospital, The Second Affiliated Hospital of Guangzhou University of Chinese Medicine, Guangdong Provincial Hospital of Chinese Medicine, Guangdong Provincial Academy of Chinese Medical Sciences, Guangzhou, Guangdong, China; 5 Department of Mammary, Yueyang Hospital of Integrated Traditional Chinese and Western Medicine, Shanghai University of Traditional Chinese Medicine, Shanghai, China; 6 Department of Breast Surgery, The First Hospital of Hunan University of Chinese Medicine, Changsha, Hunan, China; 7 Department of Breast Diseases, Jiangsu Province Hospital of Chinese Medicine, The Affiliated Hospital of Nanjing University of Chinese Medicine, Nanjing, Jiangsu, China; 8 Department of Breast Surgery, The First Affiliated Hospital of Zhejiang Chinese Medical University, Zhejiang Provincial Hospital of Chinese Medicine, Hangzhou, Zhejiang, China; 9 Department of Breast, Chongqing Traditional Chinese Medicine Hospital, Chongqing, Sichuan, China; 10 Breast Department, Sanya Maternal and Child Health Hospital, Shanghai Children’s Medical Center, Sanya, Hainan, China; 11 Guangdong Provincial Key Laboratory of Clinical Research on Traditional Chinese Medicine Syndrome, Guangdong Provincial Hospital of Chinese Medicine, Guangzhou, Guangdong, China; 12 Postdoctoral Research Center, Guangdong Provincial Hospital of Chinese Medicine, Guangzhou, Guangdong, China

**Keywords:** Chinese medicine syndrome, diagnostic criteria, early breast cancer, multicenter clinical study, standardization of clinical practice

## Abstract

**Background:**

Breast cancer is the most common cancer among women. Chinese herbal medicine, which is based on accurate Chinese medicine (CM) syndrome diagnosis, plays a vital role during cancer treatment. This study aimed to validate the established CM syndrome diagnostic criteria for early breast cancer, enhancing their application in clinical settings.

**Methods:**

A multicenter prospective clinical study was conducted to collect epidemiological data on CM syndromes. Two attending doctors from the CM breast department performed syndrome differentiation using established diagnostic criteria and compared it to two clinical experts with associate senior professional titles. Metrics such as sensitivity, specificity, and accuracy were utilized to assess the validity of the diagnostic test.

**Results:**

A total of 641 eligible cases were enrolled from June 2022 to October 2023 from seven hospitals in China. The sensitivity rates for all syndromes ranged from 70.37% to 92.00%, with the highest rate for Spleen and Stomach disharmony in the postoperative stage. Specificity varied from 86.84% to 98.89%, with most criteria exceeding 90%. Overall accuracy of the diagnostic criteria was between 84.25% and 94.45%. Positive predictive values ranged from 72.22% to 98.21%, while negative predictive values spanned from 79.25% to 98.82%, with most syndromes above 80%. The concordance rate for diagnostic criteria ranged from 90.91% to 96.45%, with Kappa values between 0.747 and 0.926.

**Conclusions:**

This study demonstrated that the diagnostic criteria exhibit high reliability, confirming the validity of CM syndrome diagnostic criteria among different treatment stages for early breast cancer and contributing to standardizing clinical practice.

## 1 Introduction

Breast cancer is the most common malignancy and a major cause of cancer-related deaths among women (1, 2). Research has indicated that breast cancer patients turn to complementary medicine to relieve side effects from cancer treatments and improve their quality of life (3–5). Chinese herbal medicine (CHM) is a frequently used form of complementary medicine, with its effectiveness dependent on accurate Chinese medicine (CM) syndrome differentiation (6). As such, there is a pressing need to standardize the CM syndrome differentiation and treatment of breast cancer to enhance therapeutic outcomes in clinical settings. An essential step in advancing the understanding of early breast cancer CM syndromes involves establishing a standard for CM syndrome differentiation and quantifying as well as standardizing the syndrome differentiation process.

In our clinical practice, we noted variations in CM syndromes in response to different treatments for breast cancer and pointed out the differentiation of CM syndromes across various treatment stages (7). Researchers have also indicated that syndrome differentiation by treatment stage in breast cancer exemplifies the integration of disease and syndrome concepts (8). As such, we further utilized a systematic review (9), a clinical study (10), and a Delphi study (11) to establish diagnostic criteria for CM syndrome differentiation among different treatment stages of early breast cancer. This diagnostic criterion clearly defines primary and secondary CM syndrome diagnostic conditions through the comprehensive use of multiple statistical methods, data mining, and expert consultation meetings. Clinical validation is crucial in assessing the clinical applicability of CM syndrome research findings. However, no confirmatory tests have been conducted in published studies on breast cancer CM syndrome, leading to limited credibility and acknowledgment of the research outcomes.

To rigorously verify the diagnostic efficacy of these criteria, this study conducted a prospective, multicenter, large-sample clinical investigation to gather clinical case information on early breast cancer. Diagnostic evaluation tests were then performed to offer evidence-based medical support in clinical practice and enhance the credibility of stage syndrome differentiation.

## 2 Materials and methods

This study was approved by the Ethics Committee of Guangdong Provincial Hospital of Chinese Medicine (approval no. ZE2022-142-01) before recruitment commenced. Other centers received ethics approval from their centers. All participants provided written informed consent. This study was registered with the Chinese Clinical Trial Registry under the registration number ChiCTR2200061022 on 15 June 2022.

### 2.1 Inclusion and exclusion criteria

Patients were selected based on the inclusion and exclusion criteria. The inclusion criteria included (1): histologically confirmed primary invasive breast cancer (Stage I–III, no distant metastases) (2); female patients aged 18 years or older (3); an Eastern Cooperative Oncology Group (ECOG) performance status of 0–2; and (4) ability to understand the study and provide written informed consent. The exclusion criteria were (1): the presence of accompanying severe diseases, such as cardio-cerebrovascular or psychological disease (2); undergoing neoadjuvant therapy or receiving concurrent treatments among chemotherapy, radiation therapy, and endocrine treatment (3); complications arising from breast cancer treatment, such as postoperative wound infection, severe lymphoedema, neutropenic fever or radiation pneumonia; and (4) pregnant or breastfeeding women.

### 2.2 Study procedure and data collection

Based on a previously published clinical study used to develop diagnostic criteria (10), the case report form for this validation study was designed according to the research review of literature and textbooks. The form encompasses several key sections, including patient basic information, breast cancer history, as well as symptoms and signs.

Patients were screened to assess whether they met the inclusion and exclusion criteria. Researchers thoroughly explained the study’s objectives and procedures to ensure complete understanding and informed consent from the patients. Additionally, participants were informed that they would receive a questionnaire survey at each stage of treatment (including preoperative, postoperative, chemotherapy, radiotherapy, and endocrine therapy). Once the patient signed the informed consent form, the first questionnaire survey began and subsequent treatments were tracked. Follow-up questionnaires were administered upon each return to the hospital for treatment. In total, each patient completed up to five surveys corresponding to each of the treatment phases.

Researchers at each hospital underwent training on the standard operating procedures, which included a comprehensive explanation of the study’s methodology and detailed instructions on obtaining informed consent and accurately completing the case report form. This training ensured that all researchers thoroughly understood the procedures and could implement them correctly. Finally, the syndrome name of each cluster was determined in accordance with the Clinic Terminology of Traditional Chinese Medical Diagnosis and Treatment–Syndromes (12), following thorough discussion with the research team.

### 2.3 Sample size calculation

The sample size estimate for this diagnostic test should be based on the assumed test’s sensitivity and specificity, as well as permissible levels of α error and test error. The formula used is: $n_{1}=\frac{\left(z_{\alpha }^{2})s_{n}(1-s_{n}\right)}{\Delta^{2}}$, $n_{2}=\frac{\left(z_{\alpha }^{2})s_{p}(1-s_{p}\right)}{\Delta^{2}}$, where $n_{1}$ and $n_{2}$ are the numbers of samples needed for the test standard and reference, respectively. Here, Δ  represents the allowable error, $s_{n}$ is the sensitivity, and $s_{p}$ is the specificity. Setting α=0.05, Δ=0.10, and we calculated Zα=1.96. With estimated sensitivity and specificity at 0.90, the required sample size for each syndrome type in the diagnostic test is calculated. Given that the study includes nine different syndromes, the estimated sample size needed is 70 samples per syndrome (in total nine syndrome according to previous Delphi study) (11), resulting in 630 cases overall. The target number of cases per site was derived from each center’s 2021 early-stage breast cancer records.

### 2.4 Diagnostic test evaluation analysis

After collecting patient data, a diagnostic test evaluation was performed. The authenticity and clinical application value evaluation of diagnostic tests were divided into two groups: the reference standard group and the test standard group.

The diagnostic results are assessed by two physicians with associate senior titles based on their experience, and they are valid as a reference standard when their results are consistent. Initially, a preliminary trial is conducted to evaluate the consistency of results between the two doctors. They first differentiate 20 randomly selected cases. After a week, they repeat the process for the same cases, and their consistency is analyzed. A Kappa value above 0.4, calculated using SPSS software, indicates high consistency, allowing formal differentiation to proceed. The test standard group comprises two CM attending physicians specialized in breast cancer, performing syndrome differentiation based on diagnostic criteria (see **Supplementary File**) established according to previous studies (9–11). Similarly, these two doctors conducted preliminary tests using the clinical data of 20 selected patients. The formal study was initiated once the Kappa value reached 0.7 or higher, indicating sufficient consistency between the doctors’ assessments.

SPSS statistical software (IBM SPSS Statistics 26.0) was utilized for statistical analysis in this study. Descriptive statistics were applied to analyze the demographic information of the patients. Shapiro-Wilk tests were conducted to assess the normal distribution of continuous variables. Measurement data are presented using maximum, minimum, average values, and standard deviation. Additionally, rates, proportions, and frequencies were used to express categorical data.

Sensitivity measures the proportion of correctly identified positive patients, with higher sensitivity indicating fewer missed diagnoses. Specificity measures the proportion of correctly identified negative patients, with higher specificity indicating fewer false positives. Accuracy represents the proportion of true positive and true negative results out of all cases, reflecting the consistency between diagnostic tests and expert evaluations. The Youden index, calculated as sensitivity plus specificity minus one, assesses the overall effectiveness of a diagnostic test. Likelihood ratios reflect the sensitivity and specificity by comparing the probability of test results in patients versus non-patients.

Predictive values assess patient prevalence based on diagnostic test results. The positive predictive value indicates the proportion of true positives among those diagnosed positive, while the negative predictive value reflects the proportion of true negatives among those diagnosed negative. Higher values enhance the diagnostic standard’s discriminatory power and clinical relevance. The Kappa value measures diagnostic consistency: values above 0.75 indicate good consistency, values between 0.4 and 0.75 suggest moderate consistency, and values below 0.4 denote poor consistency. A higher Kappa value signifies more reliable diagnostic criteria.

## 3 Results

**Figure 1:**
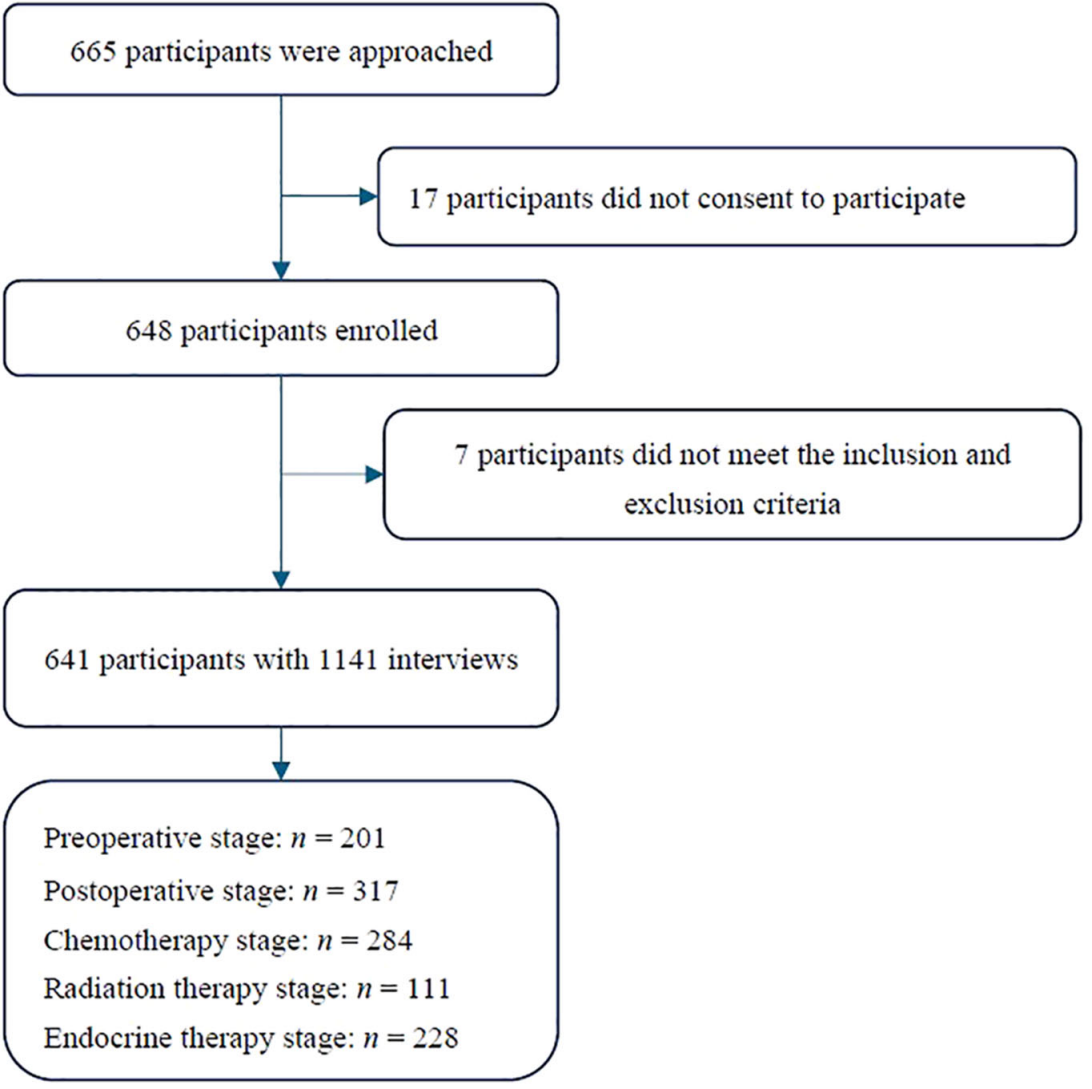
Flowchart of the participant inclusion. *n* represent the number of interviews.

**Figure 2:**
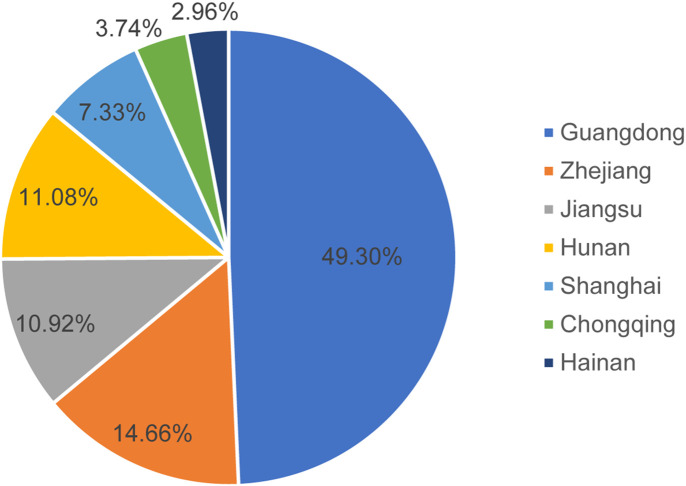
Distribution of enrolled cases across participating centers.

This study was carried out in multiple centers from June 2022 to October 2023, including the hospitals located in South China (Guangdong, Hainan), East China (Shanghai, Jiangsu, Zhejiang), Central China (Hunan), and Southwest China (Chongqing). A total of 665 patients were initially approached for participation in this study. Among them, 17 patients did not provide informed consent, and seven were excluded because they did not meet the inclusion criteria. Ultimately, 641 eligible patients were included in the study. A total of 1141 case report forms were collected, as illustrated in **Figure 1**; the distribution of cases across centers is detailed in **Figure 2**.

### 3.1 Patient characteristics

The demographic characteristics of the patients are presented in **Table 1**. The ages of the participants ranged from 25 to 88 years. Most patients were married, making up 93.76% of the total. The pathological stages were stage I (34.63%) and stage II (43.99%), with over 80% of patients diagnosed with invasive ductal carcinoma.

**Table 1:** Participant Characteristics (*N* = 641).

Demographic Characteristics	Participants (*N* = 641)	Clinical Characteristics	Participants (*N* = 641)
**Age (years)**		**ECOG performance status**	
Median	53	0 score	401 (62.56%)
Range	25–88	1 score	230 (35.88%)
P_25_, P_75_	45, 60	2 score	10 (1.56%)
**Height (cm)**		**Surgery category**	
Median	158	Conservation surgery	256 (39.94%)
Range	144–176	Mastectomy	385 (60.06%)
P_25_, P_75_	155, 162	**Pathological stage**	
**Weight (kg)**		Stage 0	41 (6.40%)
Median	57	Stage I	222 (34.63%)
Range	37–88	Stage II	282 (43.99%)
P_25_, P_75_	52, 63	Stage III	96 (14.98%)
**BMI (kg/m^2^)**		**Histologic type**	
Median	22.68	Ductal	37 (5.77%)
Range	15.61–41.66	Invasive ductal	527 (82.22%)
P_25_, P_75_	20.96, 25.08	Invasive lobular	14 (2.18%)
**Marriage status**		Other	63 (9.83%)
Single	22 (3.43%)		
Married	601 (93.76%)		
Divorced	10 (1.56%)		
Widowed	8 (1.25%)		

### 3.2 Diagnostic test evaluation results

The self-differentiation results of two doctors in the pre-experiment reference standard group were consistent. The Kappa values for the consistency tests for two doctors in the reference standard group were 0.725 and 0.673, respectively, exceeding the 0.4 threshold, indicating moderate consistency. Additionally, after conducting a formal consistency test for the syndrome differentiation results of the two doctors, a Kappa value of 0.535 was obtained, further supporting the moderate consistency of their assessments.

The syndrome differentiation results of the two doctors in the test standard group were tested for consistency, and the Kappa value was 0.837, which is greater than 0.7, indicating a high level of agreement. In cases where there were discrepancies in the syndrome differentiation results during the pre-experiment phase, the two doctors engaged in discussions and negotiations to reach a consensus on the syndrome differentiation before proceeding with the formal differentiation process.

### 3.3 Evaluation of diagnostic criteria

The reliability of the diagnostic criteria and the evaluation results are detailed in **Table 2**. Sensitivity rates for all identified syndromes ranged between 70.37% and 92.00%. Notably, the sensitivity of the Spleen and Stomach disharmony in the postoperative stage was highest, suggesting its effectiveness in accurately identifying patients. Specificity across the criteria varied from 86.84% to 98.89%. Except for the dual deficiency of *qi* and *yin* in the endocrine therapy stage, the specificity of other syndrome diagnostic criteria exceeded 90%, demonstrating a notably low rate of misdiagnosis across the five treatment stages.

**Table 2:** The authenticity of the diagnostic criteria and the evaluation results of clinical application value.

Stage	Syndrome	Sensitivity (%)	Specificity (%)	Youden index	Accuracy (%)	Positive likelihood ratio	Negative likelihood ratio	Positive predictive value (%)	Negative predictive value (%)
**Preoperative (*N* = 112)**	Liver stagnation with congealing phlegm	84.62%	97.87%	0.82	90.18%	39.77	0.16	98.21%	82.14%
	Blood stasis with phlegm	76.47%	97.89%	0.74	94.64%	36.32	0.24	86.67%	95.88%
	Disharmony of thoroughfare and conception vessels	70.37%	95.29%	0.66	89.29%	14.95	0.31	82.61%	91.01%
**Postoperative (*N* = 197)**	Dual deficiency of *qi* and Blood	80.18%	97.67%	0.78	87.82%	34.48	0.20	97.80%	79.25%
	Dual deficiency of *qi* and *yin*	79.31%	95.68%	0.75	90.86%	18.37	0.22	88.46%	91.72%
	Spleen and Stomach disharmony	92.00%	97.09%	0.89	96.45%	31.65	0.08	82.14%	98.82%
**Chemotherapy (*N* = 132)**	Dual deficiency of *qi* and Blood	73.81%	98.89%	0.73	90.91%	66.43	0.26	96.88%	89.00%
	Spleen and Kidney deficiency	85.25%	92.96%	0.78	89.39%	12.10	0.16	91.23%	88.00%
	Spleen and Stomach disharmony	89.66%	97.09%	0.87	95.45%	30.78	0.11	89.66%	97.09%
**Radiation therapy (*N* = 83)**	Dual deficiency of *qi* and *yin*	73.33%	98.48%	0.72	93.83%	48.40	0.27	91.67%	94.20%
	*Yin* deficiency with fluid depletion	81.25%	92.54%	0.74	90.36%	10.89	0.20	72.22%	95.38%
	*Yin* deficiency with fire toxin	88.00%	93.94%	0.82	90.36%	14.52	0.13	95.65%	83.78%
**Endocrine therapy (*N* = 127)**	Disharmony of thoroughfare and conception vessels	75.00%	95.33%	0.70	92.13%	16.05	0.26	75.00%	95.33%
	Dual deficiency of *qi* and *yin*	80.39%	86.84%	0.67	84.25%	6.11	0.23	80.39%	86.84%
	Spleen and Kidney deficiency	72.55%	96.10%	0.69	86.72%	18.62	0.29	92.50%	84.09%

Additionally, the overall accuracy of these criteria was observed to range from 84.25% to 94.45%. The positive predictive value for all diagnosed syndromes varied from 72.22% to 98.21%, with the positive predictive value of the remaining syndromes exceeding 80% except for the syndrome of *yin* deficiency and fluid depletion in the radiotherapy stage and disharmony of thoroughfare and conception vessels in the endocrine therapy stage. The negative predictive value ranged from 79.25% to 98.82%. Except for dual deficiency of *qi* and Blood, the negative predictive value of the remaining syndrome was greater than 80%.

The reliability evaluation results for the diagnostic criteria are presented in **Table 3**. The concordance rate for these criteria ranged from 90.91% to 96.45%, with the lowest rate observed for Spleen and Stomach disharmony during the chemotherapy stage and the highest for the dual deficiency of *qi* and *yin* in the postoperative stage. Kappa values, which measure inter-rater agreement, ranged from 0.747 to 0.926. The Kappa value for Blood stasis with phlegm in the preoperative period was 0.747, indicating moderate reliability for this syndrome’s diagnostic criteria. In contrast, the Kappa value for the other syndromes exceeded 0.750, suggesting good reliability for their diagnostic criteria.

**Table 3:** Reliability evaluation results of the diagnostic criteria.

Stage	Syndrome	Doctor C	Doctor D		Coincidence rate (%)	Kappa value
			Positive	Negative		
**Preoperative (*N* = 112)**	Liver stagnation with congealing phlegm	Positive	56	9	91.07%	0.821
		Negative	1	46
	Blood stasis with phlegm	Positive	15	5	92.86%	0.747
		Negative	3	89
	Disharmony of thoroughfare and conception vessels	Positive	23	2	95.54%	0.873
		Negative	3	84
**Postoperative (*N* = 197)**	Dual deficiency of *qi* and Blood	Positive	91	0	93.40%	0.869
		Negative	13	93
	Dual deficiency of *qi* and *yin*	Positive	52	3	96.45%	0.912
		Negative	4	138
	Spleen and Stomach disharmony	Positive	28	12	93.40%	0.773
		Negative	1	156
**Chemotherapy (*N* = 132)**	Dual deficiency of *qi* and Blood	Positive	32	2	94.70%	0.865
		Negative	5	93
	Spleen and Kidney deficiency	Positive	57	4	93.18%	0.863
		Negative	5	66
	Spleen and Stomach disharmony	Positive	29	8	90.91%	0.767
		Negative	4	91
**Radiation therapy (*N* = 83)**	Dual deficiency of *qi* and *yin*	Positive	14	3	93.98%	0.812
		Negative	2	64
	*Yin* deficiency with fluid depletion	Positive	18	1	93.98%	0.838
		Negative	4	60
	*Yin* deficiency with fire toxin	Positive	46	2	96.39%	0.926
		Negative	1	34
**Endocrine therapy (*N* = 127)**	Disharmony of thoroughfare and conception vessels	Positive	20	8	92.13%	0.752
		Negative	2	97
	Dual deficiency of *qi* and *yin*	Positive	51	1	95.28%	0.903
		Negative	5	70
	Spleen and Kidney deficiency	Positive	40	6	92.91%	0.844
		Negative	3	78

## 4 Discussion

In this multicenter prospective clinical study, we collected epidemiological data on CM syndromes to evaluate diagnostic tests, specifically assessing the diagnostic efficacy of CM syndrome differentiation criteria for early breast cancer. To the best of our knowledge, this is the first publication that verifies the CM syndrome diagnostic criteria in the context of breast cancer. This study contributes significantly to the standardization of clinical practice and offers a crucial reference for future investigations into the diagnosis of breast cancer syndromes.

The primary goal of establishing diagnostic criteria for CM syndrome is to facilitate their application in clinical practice and enhance standardization (13, 14). The scientific validation of these criteria is essential for their adoption and critical in assessing their validity and reliability. For instance, Luo (15) conducted a diagnostic test evaluation on Blood stasis syndrome, demonstrating high diagnostic effectiveness and greater reliability. Similarly, Zhou (16) evaluated the diagnostic threshold for coronary heart disease’s phlegm and dampness syndrome, improving the criteria’s diagnostic efficacy, operability, and repeatability. Despite these advances, many studies in CM syndrome research lack further validation (17), undermining the authority and recognition of their findings. This issue also pertains to breast cancer syndrome research (18–20).

Consequently, this study performs diagnostic tests on the newly established criteria for early breast cancer stage differentiation, addressing previous limitations and confirming the criteria’s effectiveness and enhanced scientific credibility.

The results demonstrate a high level of reliability in the diagnostic standards employed for evaluating the syndromes. Sensitivity and specificity are essential metrics in assessing diagnostic accuracy (21), based on the test’s inherent attributes. Sensitivity indicates the ability to avoid missed diagnoses, while specificity relates to reducing misdiagnoses. It is crucial to evaluate both metrics when analyzing diagnostic tests. However, relying solely on sensitivity and specificity may not provide a complete assessment; additional factors like accuracy and the Youden index should also be taken into account. The Youden index and likelihood ratios are composite indices that reflect both sensitivity and specificity (22). The positive and negative predictive values are significantly influenced by the disease’s prevalence (23). With constant sensitivity and specificity, the positive predictive value increases as prevalence rises, whereas the negative predictive value decreases. Thus, in cases where the disease prevalence is low, even highly sensitive and accurate diagnostic criteria may yield a low positive predictive value.

Conducting multicenter clinical research is the optimal approach for collecting comprehensive diagnostic data (24), but should be careful about data interpretation bias (25). Wang (26) conducted a multicenter cross-sectional study on damp-heat syndrome in diabetic coronary heart disease, including a broad array of research subjects to ensure representativeness. Currently, many CM syndrome studies, including those on breast cancer, are limited to one research institution or one research region (18, 27, 28). This limitation leads to sample selection bias and inadequate sample sizes, compromising the objectivity and evidential strength of the research (29). Additionally, some multicenter studies are restricted to specific areas, lacking nationwide representation. To address these issues, this study undertakes a large-scale, multicenter clinical study across various regions to gather epidemiological data, thereby enhancing the reliability and applicability of the research findings.

In addition, due to objective differences in the scale and patient flow of the participating centers, there were disparities in the number of cases ultimately contributed by each center, resulting in an inherent imbalance in the data. The International Council for Harmonization of Technical Requirements for Pharmaceuticals for Human Use (ICH) E9 (Statistical Principles for Clinical Trials) guideline suggests that exploring center effects is necessary when the sample size in each center is sufficient. In this diagnostic test study, where inter−center sample sizes varied considerably and the primary aim was to verify the generalizability of the diagnostic criteria, we focused on evaluating the overall performance of the criteria in the pooled population from all centers. Therefore, we ultimately did not perform an inter−center heterogeneity analysis.

The scientific establishment of diagnostic criteria for CM syndromes constitutes a foundational element in realizing the standardization and regularization of CM diagnosis and treatment. Historically, the absence of well-defined and universally accepted protocols for the development of these criteria has significantly impeded their formulation and subsequent dissemination. In 2023, the World Federation of Chinese Medicine Societies addressed this gap by drafting the “Guidelines on establishing diagnostic criteria of Chinese medicine syndromes” (30), which was informed by the preexisting technical framework for CM syndrome standard development. The guidelines delineate a comprehensive development process that encompasses literature review, expert consultation, clinical investigation, formulation of syndrome diagnostic criteria, and validation. Our research project has already undertaken relevant preliminary studies and is currently advancing the final phase of clinical validation in strict adherence to contemporary standard research methodologies, thereby exemplifying a robust paradigm for the development of CM syndrome standards. Further, the field of CM syndrome research remains replete with areas of inquiry that warrant further exploration. These include the objective diagnosis of CM syndromes, the elucidation of their scientific connotations, and the integration of CM syndromes with artificial intelligent (31, 32). These emerging frontiers of research are poised to catalyze our continued advancement in this domain.

Some limitations should be acknowledged. The lack of a recognized gold standard for reference and reliance on expert experience introduces an inevitable subjective bias, and this shortcoming also mirrors the broader methodological constraints currently faced in validating CM syndromes. Further, the lack of clear decision thresholds for each diagnostic test index prevents objective and accurate assessment of the diagnostic criteria. In this study, patients who did not receive endocrine therapy were excluded from enrollment. This exclusion underscores a gap in CM syndrome research for this population, limiting the diagnostic criteria’s applicability. The suboptimal diagnostic performance observed for certain syndromes may be attributed to their relatively small sample sizes, which also precluded further subgroup analysis. This underscores the need to enlarge the overall sample and achieve a more balanced distribution across syndromes in future work, thereby enhancing statistical power and robustness.

## 5 Conclusion

This multicenter prospective clinical study aimed to validate the diagnostic criteria for CM syndrome among different treatment stages in early breast cancer. The study collected substantial clinical data and performed a diagnostic evaluation test on the criteria for syndrome diagnosis. The results demonstrated high reliability in the diagnostic standards. Ultimately, the diagnostic test confirmed the authenticity, reliability, and clinical application value of CM syndrome differentiation criteria for early breast cancer. This research addressed a gap in verifying CM syndrome diagnostic criteria for breast cancer and significantly contributed to standardizing clinical practice. Further, this study lays a valuable foundation for future studies and enhances the understanding and application of CM syndrome in the context of breast cancer.

## Data Availability

The original contributions presented in the study are included in the article/**Supplementary Material**. Further inquiries can be directed to the corresponding authors.

## References

[1] Xia C Dong X Li H Cao M Sun D He S . Cancer statistics in China and United States, 2022: profiles, trends, and determinants. Chin Med J (Engl). (2022) 135:584–90. doi: 10.1097/cm9.0000000000002108, PMID: 35143424 PMC8920425

[2] Siegel RL Kratzer TB Giaquinto AN Sung H Jemal A . Cancer statistics, 2025. CA Cancer J Clin. (2025) 75:10–45. doi: 10.3322/caac.21871, PMID: 39817679 PMC11745215

[3] Guerra-Martín MD Tejedor-Bueno MS Correa-Casado M . Effectiveness of complementary therapies in cancer patients: a systematic review. Int J Environ Res Public Health 18(3). (2021) 18:1017. doi: 10.3390/ijerph18031017, PMID: 33498883 PMC7908482

[4] Ng JY Sahak H Lau SKC . A systematic review and quality assessment of breast cancer clinical practice guidelines providing complementary and alternative medicine recommendations. Curr Oncol Rep. (2021) 23:112. doi: 10.1007/s11912-021-01109-8, PMID: 34342715

[5] Han G Lee YS Jang HJ Kim SY Lee YJ Ha IH . Symptom management and quality of life of breast cancer patients using acupuncture-related therapies and herbal medicine: a scoping review. Cancers (Basel). (2022) 14:4683. doi: 10.3390/cancers14194683, PMID: 36230606 PMC9564317

[6] Zhou X Li CG Chang D Bensoussan A . Current status and major challenges to the safety and efficacy presented by Chinese herbal medicine. Medicines. (2019) 6:14. doi: 10.3390/medicines6010014, PMID: 30669335 PMC6473719

[7] Breast Disease Prevention and Treatment Cooperation Committee of the China Association of Chinese Medicine, Standards for syndrome differentiation of mammary cancer in different stages. Shanghai J Traditional Chin Med. (2010) 1):4–5. doi: 10.16305/j.1007-1334.2010.01.020

[8] Wu R Dong C . Research progress of traditional Chinese medicine in the treatment of breast cancer under stages syndrome differentiation. Chin Med Modern Distance Educ China. (2018) 16:139–42. doi: 10.3969/j.issn.1672-2779.2018.18.058

[9] Guo Q Chen Q Xue CC Zhang AL Coyle ME . Chinese medicine syndromes and stages of early breast cancer: hierarchical cluster analysis and implication for clinical practice. J Altern Complement Med. (2021) 27:904–14. doi: 10.1089/acm.2021.0055, PMID: 34076505

[10] Guo Q Coyle ME Zhang AL Xue X Bian W Song A . Chinese medicine syndrome differentiation for early breast cancer: a multicenter prospective clinical study. Front Oncol. (2022) 12:914805. doi: 10.3389/fonc.2022.914805, PMID: 35875101 PMC9300931

[11] Guo Q Zhang AL Xue CC Coyle ME Chen Q . A Delphi study of expert consensus on Chinese medicine syndrome differentiation and herbal use for early breast cancer. Integr Cancer Ther. (2023) 22:15347354231204008. doi: 10.1177/15347354231204008, PMID: 37799023 PMC10557418

[12] State Administration for Market Regulation, National Standardization Adiministration . Clinic Terminology of Traditional Chinese Medical Diagnosis and Treatment–Part 2: syndromes/patterns (2021).

[13] Tao J Yuan B Wang S . Technical methods and application status of traditional Chinese medicine syndrome research. China J Traditional Chin Med Pharm. (2018) 33:2982–5.

[14] Shi Y Wu B Li Y . Progress and thought on standardization of traditional Chinese medicine syndrome. Chin Med Modern Distance Educ China. (2019) 17:123–5. doi: 10.3969/j.issn.1672-2779.2019.17.053

[15] Luo J Wang A Zhao W Che F Fen Q Yi D . Practical diagnostic criterion of Blood stasis syndrome: introduction, reliability, and validity. Chin J Integr Med. (2015) 35:950–6. doi: 10.7661/CJIM.2015.08.0950, PMID: 26485909

[16] Zhou C Yang Y Wang C Jiang L Zhang X Zhao F . Diagnostic efficacy evaluation and threshold revision of clinical diagnostic criteria for phlegm-dampness syndrome of coronary heart disease. J Basic Chin Med. (2021) 27:1114–8. doi: 10.19945/j.cnki.issn.1006-3250.2021.07.019

[17] Chun L Xie Y Zhao H Li J . The literature analysis of normative development of traditional Chinese medicine syndrome diagnosis. J Traditional Chin Med. (2021) 62:169–72. doi: 10.13288/j.11-2166/r.2021.02.016

[18] Li C . Clinical study on traditional Chinese medicine syndrome differentiation during chemotherapy for breast cancer. Shanghai University of Traditional Chinese Medicine (2021). doi: 10.27320/d.cnki.gszyu.2021.000687

[19] Zhao N . Study on distribution and pattern of TCM syndromes in breast cancer patients receiving adjuvant endocrine therapy. Shandong University of Traditional Chinese Medicine (2018).

[20] Tang X . Study on the patterns of traditional Chinese medicine syndrome treatment for adverse reactions after endocrine therapy for breast cancer. Hunan University of Chinese Medicine (2020).

[21] Bolboacă SD . Medical diagnostic tests: a review of test anatomy, phases, and statistical treatment of data. Comput Math Methods Med. (2019) 2019:1891569. doi: 10.1155/2019/1891569, PMID: 31275427 PMC6558629

[22] Samawi H Chen DG Yin J Alsharman M . Performance of diagnostic tests based on continuous bivariate markers. J Appl Stat. (2024) 51:497–514. doi: 10.1080/02664763.2022.2137478, PMID: 38414650 PMC10898274

[23] Parikh R Mathai A Parikh S Chandra Sekhar G Thomas R . Understanding and using sensitivity, specificity and predictive values. Indian J Ophthalmol. (2008) 56:45–50. doi: 10.4103/0301-4738.37595, PMID: 18158403 PMC2636062

[24] Patil DJ Vyas T Kataria APS Rajput R Ashem A Kumar M . Multi-centric clinic trials in evidence-based research-a narrative review on the Indian scenario. J Family Med Prim Care. (2023) 12:863–7. doi: 10.4103/jfmpc.jfmpc_2257_22, PMID: 37448913 PMC10336936

[25] Jo D . The interpretation bias and trap of multicenter clinical research. Korean J Pain. (2020) 33:199–200. doi: 10.3344/kjp.2020.33.3.199, PMID: 32606263 PMC7336343

[26] Wang H Huang S Huang X Piao S Rong X Wang C . Quantitative diagnosis research of dampness-heat syndrome with diabetic coronary heart disease. J Basic Chin Med. (2024) 30:263–8. doi: 10.19945/j.cnki.issn.1006-3250.2024.02.018

[27] Meng B . Study on the change law of TCM syndromes in breast cancer patients before and after chemotherapy. Henan University of Chinese Medicine (2022). doi: 10.27119/d.cnki.ghezc.2022.000246

[28] Shi Y . Study on TCM syndrome type distribution and related influencing factors of postoperative endocrine therapy accompanying symptoms of breast cancer based on patients’ needs. Tianjin University of Traditional Chinese Medicine (2022). doi: 10.27368/d.cnki.gtzyy.2022.000350

[29] Ma Y Sun X Nian J Yu M Wang X . Thinking and advance in traditional Chinese medicine syndromes research of breast cancer. China J Traditional Chin Med Pharm. (2018) 33:3495–7.

[30] World Federation of Chinese Medicine Societies Co-construction Collaborative Innovation Center for Chinese Medicine and Respiratory Diseases by Henan & Education Ministry of P. R. China Henan University of Chinese Medicine and The First Affiliated Hospital of Henan University of Chinese Medicine . Guideline on establishing diagnostic criteria of Chinese medicine syndromes. Chin J Evidence-Based Med. (2023) 23:993–8. doi: 10.7507/1672-2531.202303134

[31] Wang Y Shi X Li L Efferth T Shang D . The impact of artificial intelligence on traditional Chinese medicine. Am J Chin Med. (2021) 49:1297–314. doi: 10.1142/s0192415x21500622, PMID: 34247564

[32] Pan D Guo Y Fan Y Wan H . Development and application of traditional Chinese medicine using AI machine learning and deep learning strategies. Am J Chin Med. (2024) 52:605–23. doi: 10.1142/s0192415x24500265, PMID: 38715181

