# Supplementary material for: Validation of Chinese medicine syndrome differentiation in early breast cancer: a multicenter prospective clinical study
Source: Front Oncol. 2026 Feb 2;16:1652339. doi: 10.3389/fonc.2026.1652339 (PMC12907149; doi:10.3389/fonc.2026.1652339)
Supplement: Supplementary file 1 [file Table1.docx]

**Supplementary file: The Chinese medicine syndrome diagnostic criteria of early breast cancer.**

| **Stage** | **Syndrome** | **Diagnostic basis** | **Diagnostic criteria** |
| --- | --- | --- | --- |
| Preoperative | Liver stagnation with congealing phlegm | 1. Depression; 2. Dysphoria, or anger, or sighing; 3. Oppression in chest; 4. Thin and white fur, or stringy pulse. | Presence one of the options 1 and 2, combined with one of options 3 and 4, the syndrome can be diagnosed. |
|  | Blood stasis with phlegm | 1. Hard breast mass, or fixed breast mass; 2. Dim complexion; 3. Menstrual color dark purple, or menstrual blood clot; 4. Dark tongue, or tortuous sublingual vessels, or stringy pulse, or hesitant pulse. | Presence of option 1, combined with any two of options 2, 3 and 4, the syndrome can be diagnosed. |
|  | Disharmony of thoroughfare and conception vessels | 1. Hard breast mass, or breast pain; 2. Dim complexion, or chloasma; 3. Soreness and weakness of waist and knees; 4. Multiple miscarriage or termination (over 3 times); 5. Pale tongue, or thin fur, or deep and thready pulse. | Presence of option 1, combined with any two of options 2, 3, 4 and 5, the syndrome can be diagnosed. |
| Postoperative | Dual deficiency of *qi* and Blood | 1. Lassitude and lack of strength; 2. Pale complexion, or sallow complexion; 3. Speechless, or shortage of *qi*; 4. Spontaneous sweating, or profuse sweating; 5. Scant menstruation, or delayed menstruation; 6. Pale tongue, or thin fur, or thready pulse, or weak pulse. | Presence of option 1 and 2, combined with any two of options 3, 4, 5 and 6, the syndrome can be diagnosed. |
|  | Dual deficiency of *qi* and *yin* | 1. Lassitude and lack of strength; 2. Dry mouth, or dry throat; 3. Speechless or shortage of *qi*; 4. Night sweating, or spontaneous sweating; 5. Constipation; 6. Thin fur, or thready pulse, or rapid pulse. | Presence of option 1 and 2, combined with any two of options 3, 4, 5 and 6, the syndrome can be diagnosed. |
|  | Spleen and Stomach disharmony | 1. Anorexia, or belching; 2. Abdominal fullness, or loose stool; 3. Sallow complexion; 4. Vomiting, or retch; 5. Pale tongue, or pink tongue, or teeth marks tongue, or greasy fur, or slippery pulse. | Presence of option 1 and 2, combined with any one of options 3, 4 and 5, the syndrome can be diagnosed. |
| Chemotherapy | Dual deficiency of *qi* and Blood | 1. Lassitude and lack of strength, or dizziness; 2. Pale complexion, or pale lips; 3. Speechless, or shortage of *qi*; 4. Palpitation; 5. Spontaneous sweating; 6. Scant menstruation, or delayed menstruation, or amenorrhea; 7. Pink tongue, or thin tongue, or thin and white fur, or thready pulse. | Presence two of the options 1, 2 and 3, combined with any two of the options 4, 5, 6 and 7, the syndrome can be diagnosed. |
|  | Spleen and Kidney deficiency | 1. Lassitude and lack of strength; 2. Sallow complexion; 3. Nausea, or retch; 4. Abdominal fullness, or diarrhea, or loose stool, or undigested food in stool; 5. Cold limbs; 6. Soreness and weakness of waist and knees; 7. Frequent urination at night; 8. Dark tongue, or plump tongue, or teeth marks tongue, or thready pulse, or deep pulse. | Presence of option 1, combined with any three of options 2, 3, 4, 5, 6, 7 and 8, the syndrome can be diagnosed. |
|  | Spleen and Stomach disharmony | 1. Anorexia, or nausea, or vomiting, or retch; 2. Sallow complexion; 3. Acid regurgitation, or belching; 4. Abdominal fullness, or abdominal pain, or loose stool; 5. Plump tongue, or teeth marks tongue, or greasy fur, or slippery pulse. | Presence of option 1, combined with any two of options 2, 3, 4 and 5, the syndrome can be diagnosed. |
| Radiation therapy | Dual deficiency of *qi* and *yin* | 1. Lassitude and lack of strength, or shortage of *qi*; 2. Dry mouth, or dry throat; 3. Vexing heat in chest, palms and soles, or tidal fever, or spontaneous sweating; 4. Cheek redness; 5. Constipation; 6. Red tongue, or dry tongue, or thready pulse. | Presence of option 1 and 2, combined with any two of options 3, 4, 5 and 6, the syndrome can be diagnosed. |
|  | *Yin* deficiency with fluid depletion | 1. Dryness of the radiated skin, or itching of the radiated skin, or pain of the radiated skin; 2. Dry mouth, or dry throat, or pain throat; 3. Constipation; 4. Aphtha; 5. Emaciation; 6. Insomnia; 7. Red tongue, or dry tongue, or thready pulse, or thready and rapid pulse. | Presence two of the options 1, 2 and 3, combined with any two of the options 4, 5, 6 and 7, the syndrome can be diagnosed. |
|  | *Yin* deficiency with fire toxin | 1. Redness of radiated skin, or pain of the radiated skin, or ulcers of the radiated skin; 2. Aphtha; 3. Dry mouth, or dry throat, or pain throat; 4. Vexation; 5. Cheek redness; 6. Constipation; 7. Red tongue, or dry tongue, or thin fur, or rapid pulse. | Presence two of the options 1, 2 and 3, combined with any two of the options 4, 5, 6 and 7, the syndrome can be diagnosed. |
| Endocrine therapy | Disharmony of thoroughfare and conception vessels | 1. Tidal fever, or profuse sweating; 2. Chloasma; 3. Arthralgia, or soreness and weakness of waist and knees; 4. Amenorrhea; 5. Pink tongue, or red tongue, or thin fur. | Presence of option 1, combined with any two of options 2, 3, 4 and 5, the syndrome can be diagnosed. |
|  | Dual deficiency of *qi* and *yin* | 1. Lassitude and lack of strength, or shortage of *qi*, or speechless; 2. Night sweating, or spontaneous sweating, or tidal fever; 3. Dry mouth, or dry throat; 4. Cheek redness; 5. Vexation; 6. Constipation; 7. Pink tongue, or red tongue, or dry tongue, or thin tongue, or thready pulse. | Presence two of the options 1, 2 and 3, combined with any two of the options 4, 5, 6 and 7, the syndrome can be diagnosed. |
|  | Spleen and Kidney deficiency | 1. Lassitude and lack of strength, or dizziness and shortage of *qi*; 2. Sallow complexion; 3. Anorexia; 4. Aversion to cold with cold limbs; 5. Soreness and weakness of waist and knees, or arthralgia; 6. Abdominal fullness, or diarrhea, or loose stool; 7. Frequent urination at night, or large volume of clear urine; 8. Dark tongue, or plump tongue, or thready pulse, or deep pulse. | Presence of option 1, combined with any three of options 2, 3, 4, 5, 6, 7 and 8, the syndrome can be diagnosed. |
